# Analytical interpretation of hemodynamic data in patients with intracardiac shunts: Role of mathematical modeling

**DOI:** 10.1016/j.ijcchd.2026.100662

**Published:** 2026-02-22

**Authors:** Paolo Ferrero, Andrea Tonini, Giulio Valenti, Pier Paolo Bassareo, Stiljan Hoxha, Luca Dedè, Alfio Quarteroni, Konstantinos Dimopoulos

**Affiliations:** a Cardiopulmonary and vascular department. AOUI, Verona, Italy; b MOX-Modeling and Scientific Computing, Department of Mathematics, Politecnico di Milano, Milan, Italy; c Cardiac Surgery University Hospital Verona, Verona, Italy; d University College of Dublin, School of Medicine, Dublin, Ireland; e Adult Congenital Heart Centre and Centre for Pulmonary Hypertension, Royal Brompton Hospital, Part of GSTT, London, United Kingdom

**Keywords:** Shunt, Pulmonary vascular resistances, Model, Pre-tricuspid

## Abstract

**Background:**

Shunt lesions are categorized into pre- and post-tricuspid. Although it is well recognized that these two entities have a different pathophysiology, hemodynamic variables involved are still poorly understood. This paper aims to analytically appraise shunt physiology exploiting a lumped parameters mathematical model.

**Methods:**

Circulatory system was split into arterial and venous compartments, each of them being described by resistive, capacitive and inductive components. The model was modified including communication between atria and ventricles. Predicted changes in the ratio between pulmonary blood flow and systemic blood flow (Qp/Qs), obtained by manipulating pulmonary resistances (PVR) and ventricular stiffness were computed.

**Results:**

A twofold rise of pulmonary vascular resistance resulted in a significant reduction of Qp/Qs in the setting of isolated ventricular septal defect (VSD) and VSD associated with atrial septal defect (ASD) but did not produce a sizable effect in case of isolated ASD. In the model describing an isolated ASD, a similar magnitude of Qp/Qs reduction was predicted by simulating an increase of right ventricular passive elastance and relaxation time. In this type of shunt, the dependence of Qp/Qs from PVR and ventricular elastance appeared analytically linked.

**Conclusions:**

This model analytically illustrates that shunt through an ASD is minimally affected by PVRs. The marginal change of pulmonary flow produced by large variations of PVR appeared mediated by changes in right ventricular elastance. The effect of pulmonary vasodilators in patients with ASD can be concealed or enhanced by increased stiffness of the right or left ventricle, respectively.

## 1 Background

Intracardiac and extracardiac shunts occur as a result of congenital heart defects that allow communication between cardiac chambers or major vessels. The cornerstone of the hemodynamic assessment of patients with intracardiac shunts is the calculation of shunt fraction (Qp/Qs), and the measurement of the transpulmonary gradient and pulmonary flow (Qp) that allow estimation of the pulmonary vascular resistances (PVR).

From an anatomical point of view, shunt lesions are classified into pre-tricuspid, typically atrial septal defects (ASD), versus post tricuspid (ventricular septal defects, VSD), patent ductus arteriosus (PDA) or aortopulmonary window [1,2]. The direction and severity of a post-tricuspid shunt depends heavily on the size of the defect and ratio between systemic and pulmonary vascular resistance (SVR and PVR, respectively) whereas, in pre-tricuspid shunts, the diastolic properties of the ventricles play a significant role, by determining the pressure gradient between the atria.

To calculate PVR, pulmonary artery pressure (PAP), Qp and PVR are assumed to be linearly related.

This is, however, an oversimplification of the physiology of the pulmonary circulation, ignoring flow pulsatility, ventricular elastance, pulmonary artery capacitance and blood inertia. These obvious limitations can be overcome by exploiting an electric circuit analogue, analytically described by a zero-dimensional lumped parameter mathematical model, that can be used to better describe shunt physiology in different anatomical scenarios. Such models are particularly valuable, as they can be adapted to almost all anatomic variants of congenital heart disease and allow us to analytically compute the effect of simulated changes in physiological variables.

In this paper we appraise the hemodynamic variables governing shunt fraction using a modified zero-dimensional mathematical model representing an ASD, a VSD, and an ASD associated with VSD.

## 2 Methods

**Fig. 1:**
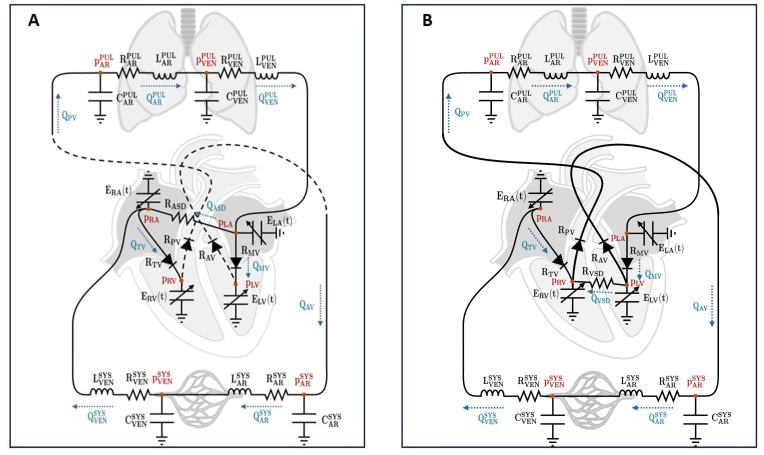
Diagram illustrating the electric analogue model representing the shunt through ASD **(panel A)** and VSD **(panel B)**. The model parameters meanings are reported in Table 1. $p_{\text{AR}}^{\text{PUL}}$: pulmonary arteries pressure; $Q_{\text{AR}}^{\text{PUL}}$: pulmonary arteries flux; $p_{\text{VEN}}^{\text{PUL}}$: pulmonary veins pressure; $Q_{\text{VEN}}^{\text{PUL}}$: pulmonary veins flux; $p_{\text{LA}}$: left atrium pressure; $R_{\text{MV}}$: mitral valve resistance; $Q_{\text{MV}}$: mitral valve flux; $p_{\text{LV}}$: left ventricle pressure; $R_{\text{AV}}$: aortic valve resistance; $Q_{\text{AV}}$: aortic valve flux; $p_{\text{AR}}^{\text{SYS}}$: systemic arteries pressure; $Q_{\text{AR}}^{\text{SYS}}$: systemic arteries flux; $p_{\text{VEN}}^{\text{SYS}}$: systemic veins pressure; $Q_{\text{VEN}}^{\text{SYS}}$: systemic veins flux; $p_{\text{RA}}$: right atrium pressure; $R_{\text{TV}}$: tricuspid valve resistance; $Q_{\text{TV}}$: tricuspid valve flux; $p_{\text{RV}}$: right ventricle pressure; $R_{\text{PV}}$: pulmonary valve resistance; $Q_{\text{PV}}$: pulmonary valve flux; $Q_{\text{ASD}}\text{:}$ blood flow across atrial septal defect; $Q_{\text{VSD}}$: blood flow across ventricular septal defect.

This is a proof of concept study aiming to provide an analytical and quantitative interpretation of pre and post-tricuspid shunts. The mathematical formulation of the model has been previously described [[3], [4], [5]]. Briefly, the cardiac chambers are modeled as pressure generators by means of time-varying elastances that mimic the periodic contraction of the cardiac chambers, incorporating the combined contribution of passive and active elastances. The circulatory system is split into several compartments, each represented by a Windkessel circuit. Systemic and pulmonary circulations are subdivided into arterial and venous compartments. The Windkessel circuits describe each compartment by means of resistors, representing the resistance to blood flow, capacitors representing vessels compliance, and inductors representing blood inertia. This model has been tailored to describe a shunt through an ASD and/or VSD (Fig. 1A and B). In the cases of VSD alone or associated with ASD, the baseline values for resistances, compliances, blood inertia and elastances are fixed (Table 1) so that, when the defects are closed, the model reproduces physiological hemodynamic values derived from general population without heart conditions, such as pressures and volumes, resulting in baseline SVR = 15 WU and PVR = 1.9 WU.

**Table 1:** Model parameters and the corresponding baseline values.

Parameter	VSD	ASD	ASD + VSD	Description
${\text{EA}}_{\text{LA}}$ [mmHg/mL]	0.2273	0.2273	0.2273	Left atrial active elastance
${\text{EB}}_{\text{LA}}$ [mmHg/mL]	0.209	0.209	0.209	Left atrial passive elastance
${\text{EA}}_{\text{LV}}$ [mmHg/mL]	3.0391	2.44	3.0391	Left ventricular active elastance
${\text{EB}}_{\text{LV}}$ [mmHg/mL]	0.10	0.018	0.10	Left ventricular passive elastance
${\text{EA}}_{\text{RA}}$ [mmHg/mL]	0.0429	0.0429	0.0429	Right atrial active elastance
${\text{EB}}_{\text{RA}}$ [mmHg/mL]	0.0636	0.0636	0.0636	Right atrial passive elastance
${\text{EA}}_{\text{RV}}$ [mmHg/mL]	0.6683	0.6683	0.6683	Right ventricular active elastance
${\text{EB}}_{\text{RV}}$ [mmHg/mL]	0.07	0.0088	0.07	Right ventricular passive elastance
$R_{\min}$ [mmHg s/mL]	0.0075	0.0075	0.0075	Minimal valve resistance
$R_{\max}$ [mmHg s/mL]	75006.2	75006.2	75006.2	Maximal valve resistance
$R_{\text{ASD}}$ [mmHg s/mL]	-	0.0031	0.0031	ASD resistance
$R_{\text{VSD}}$ [mmHg s/mL]	0.038	-	0.038	VSD resistance
$R_{\text{AR}}^{\text{SYS}}$ [mmHg s/mL]	0.588	0.699	0.588	Systemic arterial resistance
$C_{\text{AR}}^{\text{SYS}}$ [mL/mmHg]	0.96	0.671	0.96	Systemic arterial compliance
$L_{\text{AR}}^{\text{SYS}}$ [mmHg s^2^/mL]	5 · 10^−3^	5 · 10^−3^	5 · 10^−3^	Systemic arterial inertia
$R_{\text{VEN}}^{\text{SYS}}$ [mmHg s/mL]	0.352	0.66	0.352	Systemic venous resistance
$C_{\text{VEN}}^{\text{SYS}}$ [mL/mmHg]	60.0	60.0	60.0	Systemic venous compliance
$L_{\text{VEN}}^{\text{SYS}}$ [mmHg s^2^/mL]	5 · 10^−4^	5 · 10^−4^	5 · 10^−4^	Systemic venous inertia
$R_{\text{AR}}^{\text{PUL}}$ [mmHg s/mL]	0.104	0.290	0.104	Pulmonary arterial resistance
$C_{\text{AR}}^{\text{PUL}}$ [mL/mmHg]	5.0	5.0	5.0	Pulmonary arterial compliance
$L_{\text{AR}}^{\text{PUL}}$ [mmHg s^2^/mL]	5 · 10^−4^	5 · 10^−4^	5 · 10^−4^	Pulmonary arterial inertia
$R_{\text{VEN}}^{\text{PUL}}$ [mmHg s/mL]	0.0105	0.0105	0.0105	Pulmonary venous resistance
$C_{\text{VEN}}^{\text{SYS}}$ [mL/mmHg]	16.0	16.0	16.0	Pulmonary venous compliance
$L_{\text{VEN}}^{\text{PUL}}$ [mmHg s^2^/mL]	5 · 10^−4^	5 · 10^−4^	5 · 10^−4^	Pulmonary venous inertia
$T_{\text{HB}}$ [s]	0.8	0.8	0.8	Heartbeat period
$tC_{\text{LA}}$ [s]	0.79 $\cdot T_{\text{HB}}$	0.79 $\cdot T_{\text{HB}}$	0.79 $\cdot T_{\text{HB}}$	Time of left atrial contraction
$TC_{\text{LA}}$ [s]	0.11 $\cdot T_{\text{HB}}$	0.11 $\cdot T_{\text{HB}}$	0.11 $\cdot T_{\text{HB}}$	Duration of left atrial contraction
$tR_{\text{LA}}$ [s]	$tC_{\text{LA}}+TC_{\text{LA}}$	$tC_{\text{LA}}+TC_{\text{LA}}$	$tC_{\text{LA}}+TC_{\text{LA}}$	Time of left atrial relaxation
$TR_{\text{LA}}$ [s]	0.8 ${\cdot T}_{\text{HB}}$	0.8 ${\cdot T}_{\text{HB}}$	0.8 ${\cdot T}_{\text{HB}}$	Duration of left atrial relaxation
$tC_{\text{LV}}$ [s]	0.0	0.0	0.0	Time of left ventricular contraction
$TC_{\text{LV}}$ [s]	0.35 ${\cdot T}_{\text{HB}}$	0.35 ${\cdot T}_{\text{HB}}$	0.35 ${\cdot T}_{\text{HB}}$	Duration of left ventricular contraction
$tR_{\text{LV}}$ [s]	$tC_{\text{LV}}+TC_{\text{LV}}$	$tC_{\text{LV}}+TC_{\text{LV}}$	$tC_{\text{LV}}+TC_{\text{LV}}$	Time of left ventricular relaxation
$TR_{\text{LV}}$ [s]	0.4 ${\cdot T}_{\text{HB}}$	0.4 ${\cdot T}_{\text{HB}}$	0.4 ${\cdot T}_{\text{HB}}$	Duration of left ventricular relaxation
$tC_{\text{RA}}$ [s]	0.8 ${\cdot T}_{\text{HB}}$	0.8 ${\cdot T}_{\text{HB}}$	0.8 ${\cdot T}_{\text{HB}}$	Time of right atrial contraction
$TC_{\text{RA}}$ [s]	0.1 ${\cdot T}_{\text{HB}}$	0.1 ${\cdot T}_{\text{HB}}$	0.1 ${\cdot T}_{\text{HB}}$	Duration of right atrial contraction
$tR_{\text{RA}}$ [s]	$tC_{\text{RA}}+TC_{\text{RA}}$	$tC_{\text{RA}}+TC_{\text{RA}}$	$tC_{\text{RA}}+TC_{\text{RA}}$	Time of left atrial relaxation
$TC_{\text{RA}}$ [s]	0.7 ${\cdot T}_{\text{HB}}$	0.7 ${\cdot T}_{\text{HB}}$	0.7 ${\cdot T}_{\text{HB}}$	Duration of left atrial relaxation
$tC_{\text{RV}}$ [s]	0.0	0.0	0.0	Time of right ventricular contraction
$TC_{\text{RV}}$ [s]	0.3 ${\;\cdot T}_{\text{HB}}$	0.3 ${\;\cdot T}_{\text{HB}}$	0.3 ${\;\cdot T}_{\text{HB}}$	Duration of left ventricular contraction
$tR_{\text{RV}}$ [s]	$tC_{\text{RV}}+TC_{\text{RV}}$	$tC_{\text{RV}}+TC_{\text{RV}}$	$tC_{\text{RV}}+TC_{\text{RV}}$	Time of right ventricular relaxation
$TR_{\text{RV}}$ [s]	0.4 ${\cdot T}_{\text{HB}}$	0.4 ${\cdot T}_{\text{HB}}$	0.4 ${\cdot T}_{\text{HB}}$	Duration of left ventricular relaxation

Quantitative variations of Qp/Qs in different hemodynamic conditions were investigated by individually introducing PVR and ventricular compliance changes (Table 2), while keeping the other model parameters constant to their baseline values (Table 1). PVR varied from 1.9 WU, representing a baseline healthy value, up to 8.2 WU to investigate the effects of pulmonary vascular remodeling of increasing severity on pulmonary blood flow. The impact of right ventricular (RV) compliance was assessed by simulating an increase in either the RV passive elastance ($EB_{\text{RV}}$) or the relaxation time relative to the cycle length ($TR_{\text{RV}}/T_{\text{HB}}$), that have been shown to be reliable metrics of ventricular diastolic properties [6]. $EB_{\text{RV}}$ varied from its baseline up to four times the baseline value (0.28 mmHg/mL), in order to study the widest effect of increased RV stiffness. Finally, $TR_{\text{RV}}/T_{\text{HB}}$ varied between 30% and 70% of the heartbeat period, ensuring physiologically plausible relaxation durations while avoiding overlap with ventricular contraction.

**Table 2:** Ranges of variability of model parameters.

	Minimum	Maximum
PVR [mmHg s/mL]	1.9	8.2
$EB_{\text{RV}}$ [mmHg/mL]	0.0088	0.28
$T_{\text{RV}}/T_{\text{HB}}$	30%	70%

The quantitative changes of shunt flow through the ASD (Q_ASD_) and VSD (Q_VSD_) during the entire cycle length predicted by the model were also plotted.

## 3 Results

### 3.1 VSD modeling

**Fig. 2:**
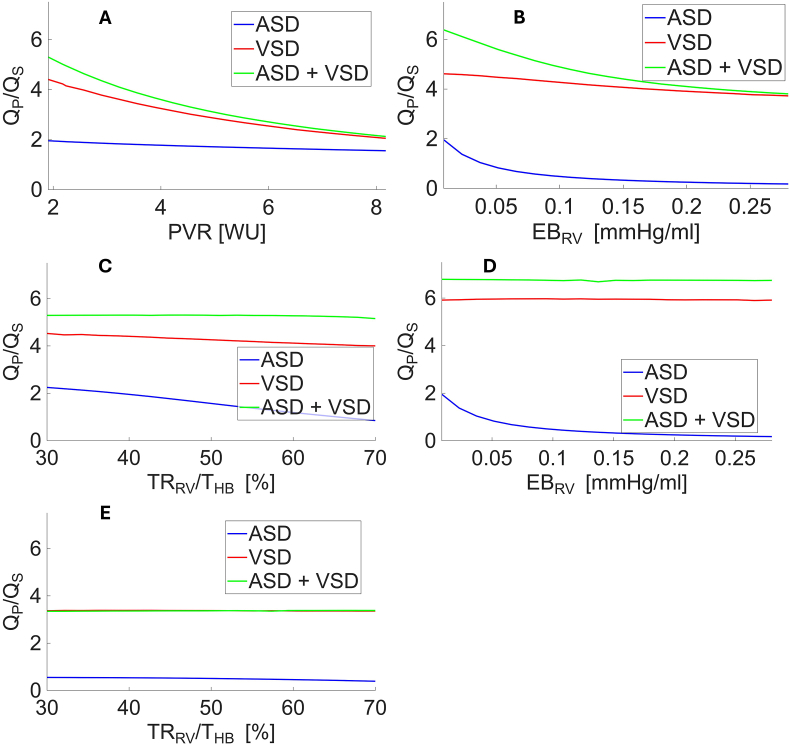
Relationship between different model parameters and Qp/Qs. **Panel A.** Relationship between pulmonary vascular resistances and Qp/Qs, with all other model parameters fixed at their baseline values. **Panel B.** Relationship between right ventricular elastance and Qp/Qs for $R_{\text{VSD}}=0.038$ mmHg s/mL, with all other model parameters fixed at their baseline values. **Panel C.** Relationship between relaxation time and Qp/Qs, with all other model parameters fixed at their baseline values. **Panel D.** Relationship between right ventricular elastance and Qp/Qs for $R_{\text{VSD}}=0.0024$ mmHg s/mL, with all other model parameters fixed at their baseline values. **Panel E.** Relationship between relaxation time and Qp/Qs for a tenfold increase of $EB_{\text{RV}}$ with respect to its baseline value, with all other model parameters fixed at their baseline values.

**Fig. 3:**
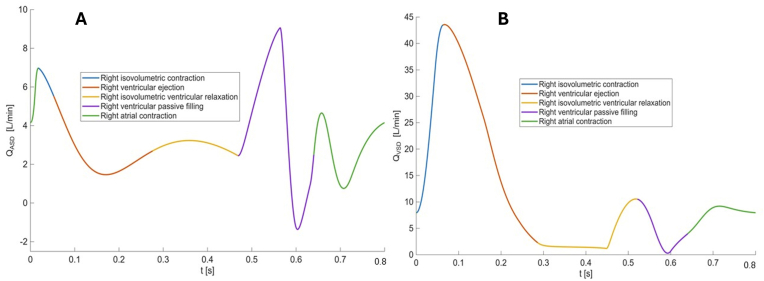
Blood flow across atrial **(panel A)** and ventricular **(panel B)** septal defect. $Q_{\text{ASD}}$: blood flow across atrial septal defect; $Q_{\text{VSD}}$: blood flow across ventricular septal defect.

In the case of a VSD, an increase in PVR (fixing all other model parameters to the baseline values) resulted in an exponential decrease in Qp/Qs (from 4.4 to 2.0, corresponding to a 53% reduction). By simulating a 2-fold increase in PVR (from 2 to 4 Wood units) a Qp/Qs decrease of approximately one unit was predicted by the model. On the other hand, changes in both $EB_{\text{RV}}$ or $TR_{\text{RV}}/T_{\text{HB}}$, had a negligible effect (Fig. 2B and C): changes in $EB_{\text{RV}}$ led to a reduction in Qp/Qs from 4.6 to 3.7 (19%), whereas variations in $TR_{\text{RV}}/T_{\text{HB}}$ resulted in a decrease in Qp/Qs from 4.5 to 4.0 (12%). Q_VSD_ predicted by the model displayed a major systolic component during the isovolumetric contraction and ejection phase and two other minor peaks coincident with ventricular relaxation and atrial contraction (Fig. 3B).

ASD: atrial septal defect, VSD: ventricular septal defect.

### 3.2 ASD modeling

Symmetrically, in the case of an isolated ASD, a PVR increase (fixing all other model parameters to the baseline values) resulted in a negligible decrease in Qp/Qs (from 2.0 to 1.6, corresponding to a 20% reduction, Fig. 2A), while $EB_{\text{RV}}$ showed the greatest effect on shunt fraction: indeed, by simulating a $EB_{\text{RV}}$ increase an exponential fall of Qp/Qs is predicted (from 2.0 to 0.2, corresponding to a 91% reduction, Fig. 2B). Similarly, an increase in $TR_{\text{RV}}/T_{\text{HB}}$ produced significant linear decrease of Qp/Qs (from 2.2 to 0.8, corresponding to a 62% reduction, Fig. 2C).

Differently from the case of VSD, shunt flow through the ASD takes place during the entire cycle length with two major peaks occurring during ventricular relaxation and atrial contraction (Fig. 3A).

### 3.3 ASD and VSD modeling

According to the model, in the case of a VSD associated with ASD, an increase in PVR (fixing all other model parameters to the baseline values) elicited a Qp/Qs decrease of a similar magnitude and with a similar course to the one observed in isolated VSD (from 5.3 to 2.1, corresponding to a 60% reduction, Fig. 2A). Passive elastance increase, again caused a sizable reduction of shunt fraction (from 6.4 to 3.8, corresponding to a 40% reduction, Fig. 2B), whereas a simulated increase of $TR_{\text{RV}}/T_{\text{HB}}$ did not produce a significant effect on Qp/Qs (from 5.3 to 5.2, corresponding to a 3% reduction, Fig. 2C).

### 3.4 Effects of variable defects sizes

According to the model, variations in defects sizes exhibit Qp/Qs trends similar to those discussed above, although with a reduced magnitude, except for the $EB_{\text{RV}}$ in the case of combined VSD and ASD. In this latter scenario, the Qp/Qs trend depends on which defect is dominant. For baseline parameters values (Fig. 2B), $R_{\text{ASD}}$ is 0.0031 mmHg s/mL and $R_{\text{VSD}}$ is 0.038 mmHg s/mL, indicating a totally negligible resistance across atrial septum and a large ventricular defect, that nonetheless limits blood flow between the ventricles. Consequently, Qp/Qs follows a trend similar to that of the isolated ASD case as a function of the $EB_{\text{RV}}$. By decreasing $R_{\text{VSD}}$ (thereby increasing the VSD size) to a negligible value, the Qp/Qs trend in the combined VSD and ASD case becomes comparable to that of the isolated VSD case, showing a reduced sensitivity of the shunt fraction to changes in $EB_{\text{RV}}$ (Fig. 2D).

### 3.5 Effects of variable right ventricular passive elastance

No significant effects were observed when varying the RV passive elastance on the relationship between Qp/Qs and either the pulmonary resistance or the RV relaxation time, except in the case of extreme variations. Introducing a tenfold increase in $EB_{\text{RV}}$ into the cardiocirculatory model makes the RV markedly stiffer and Qp/Qs in the isolated ASD case is no longer influenced by $TR_{\text{RV}}/T_{\text{HB}}$ (Fig. 2E).

## 4 Discussion

It has previously been shown that congenital conditions can be reliably simulated by a lumped parameter mathematical model [5]. Quantitative estimation of the effect of different hemodynamic determinants of pulmonary flow in the presence of simple or complex shunts can enhance the interpretation of invasive and non-invasive parameters and may assist clinical decision making.

In this zero-dimensional model of a VSD, isolated or associated with an ASD, a steep but non-linear relation between Qp/Qs and PVR can be demonstrated. The observed non-linear relation ultimately means that, for a given absolute increase or decrease in PVR, a greater change in Qp/Qs is expected in patients with a normal or near-normal PVR than in those with established pulmonary arterial hypertension. This observation might have some clinical relevance whenever a vasodilator test is performed. Conversely, the model predicts that, in a patient with an ASD, changes in PVR have a little impact on Qp/Qs suggesting that other mechanisms are responsible for the effect of pulmonary arterial hypertension (PAH) on shunt fraction observed in clinical practice.

Indeed, changes in $EB_{\text{RV}}$ (Fig. 3B) are more likely to influence shunt fraction in ASDs, with a negligible effect in VSDs. This is also confirmed by the model that shows a mainly systolic pattern of shunted flow through the VSD and a mainly diastolic flow through the ASD. While a rise in PVR is the cornerstone of PAH physiology, these results analytically show that PAH affects shunt fraction differently in pre-vs post-tricuspid defects. (Fig. 2A and B). Shunting through a post-tricuspid defect is directly affected by PVR while in pre-tricuspid defects, only at a later stage, RV adaptation to PVR increase induces RV hypertrophy and other changes that are likely to affect RV filling pressure and passive elastance, eventually affecting shunt fraction, less so in a VSD where the shunt is predominantly systolic.

According to this mathematical model, utilization of a pulmonary vasodilator is expected to elicit a fairly reproducible and predictable change in Qp/Qs is patients with a VSD, but less so in isolated ASDs, unless reverse RV remodeling occurs [7]. Similarly, a decrease in left ventricular compliance, e.g. as a result of ageing, uncontrolled hypertension, diabetes, obesity etc., is likely to drive an increase in shunt fraction at atrial level, a phenomenon that explains the surge of late diagnoses of ASDs (but not VSDs) after the 5th decade of life [8].

Interestingly, in the clinical scenario characterized by VSD associated with ASD the model predicts that the ‘dominant’ defect in terms of intrinsic resistances shapes of the Qp/Qs trend conditional to PVR and $EB_{\text{RV}}$, while $TR_{\text{RV}}/T_{\text{HB}}$ changes had a negligible effect. This finding can be explained by considering that the increase in relaxation time represents a milder degree of ventricular stiffness which is not able to affect the global shunt generated by the VSD component even in the presence of an ASD component.

These quantitative data show that additional hemodynamic variables, besides those considered in the conventional resistive model, govern shunt physiology. Moreover, the effect size of such variables varies across the range of PVR and according to the anatomical site of the shunt.

## 5 Limitations

This work represents a proof of concept for the application of a zero-dimensional cardiocirculatory model to investigate hemodynamic alterations resulting from septal defects. The current results depend on the baseline model parameters used for the simulations. As shown in the results section, modifications to baseline parameters (such as the size of septal defects or the stiffness of the RV) primarily affect the magnitude of Qp/Qs, although extreme parameter variations can also lead to different trends. To mitigate this issue, we selected a baseline configuration representing healthy individuals, avoiding biased toward pathological conditions and enabling a general analysis of Qp/Qs. Nonetheless, for patient-specific studies, the model parameters must be calibrated according to clinical data to provide meaningful insights into an individual's condition.

Therefore, the results should be mainly considered qualitative and we should be cautious in generalizing quantitative findings, as variability of genetic background and biological mechanisms of chronic adaptation cannot be contemplated by the model. Moreover, the outcomes depend on the adopted model, which must be validated against a cohort of patients before the results can be generalized to a broader population. Validation of the model against a patient cohort would also enable the estimation of confidence intervals for clinical relevant quantities, such as Qp/Qs, thereby providing more detailed insight into the effects of the septal defects.

The mathematical model considered here is zero-dimensional and lacks in spatial insights, as the defects geometry and flow directionality, information about wave propagation and ventricular and vascular coupling beyond passive elastance are not computed. Spatially resolved cardiocirculatory models could provide more detailed information on the effects of increased blood flow through the pulmonary circulation.

## 6 Conclusions

In this paper we analytically appraise quantitative effect of hemodynamic variables on shunt physiology produced by cardiac communications at different anatomic levels. Validated zero dimensional lumped parameters mathematical model of the human circulation can be tailored and manipulated, aiding in the interpretation of challenging cases and advancing our understanding of shunt lesions and their management.

## CRediT authorship contribution statement

**Paolo Ferrero:** Writing – original draft, Conceptualization. **Andrea Tonini:** Formal analysis, Data curation. **Giulio Valenti:** Software, Formal analysis, Data curation. **Pier Paolo Bassareo:** Writing – review & editing, Data curation, Conceptualization. **Stiljan Hoxha:** Writing – review & editing, Supervision, Conceptualization. **Luca Dedè:** Supervision, Conceptualization. **Alfio Quarteroni:** Writing – review & editing, Supervision. **Konstantinos Dimopoulos:** Writing – review & editing, Validation, Supervision, Conceptualization.

## Declaration of competing interest

The authors declare that they have no known competing financial interests or personal relationships that could have appeared to influence the work reported in this paper, other than KD serving the IJCCHD Editorial Board but had no involvement with the handling of this paper.
